# Field evaluation of personal protection methods against outdoor-biting mosquitoes in Lao PDR

**DOI:** 10.1186/s13071-018-3239-0

**Published:** 2018-12-17

**Authors:** Julie-Anne A. Tangena, Phoutmany Thammavong, Somsanith Chonephetsarath, James G. Logan, Paul T. Brey, Steve W. Lindsay

**Affiliations:** 1 Medical Entomology & Biology of Disease Vectors Laboratory, Institut Pasteur du Laos, Vientiane, Lao PDR; 2 Department of Disease Control, London School of Hygiene and Tropical Medicine, London, UK; 3 ARCTEC, London School of Hygiene and Tropical Medicine, London, UK; 4 Department of Biosciences, Durham University, Durham, UK

**Keywords:** Para-menthane-3,8-diol (PMD), Mosquito coil, Topical repellent, Permethrin-treated clothing

## Abstract

**Background:**

Protecting people outdoors against mosquito-borne diseases is a major challenge. Here we compared commercially available personal protection methods to identify the most effective method for outdoor use in northern Lao PDR.

**Methods:**

From June to August 2016 the protective efficacy of treatments were compared in a secondary forest during the afternoon and a village during the evening. Comparisons were made using a replicated Latin square design between: (i) short permethrin-treated overalls; (ii) long permethrin-treated overalls; (iii) short untreated overalls with para-menthane-3,8-diol (PMD) applied topically; (iv) short permethrin-treated overalls plus PMD applied topically; (v) short untreated overalls with metofluthrin coils in a metal casing worn on a belt; and (vi) long untreated overalls. Short untreated overalls served as the control. Cone tests were conducted on the treated and untreated fabric before and after field experiments. A questionnaire survey was used to measure social acceptability.

**Results:**

Mosquito coils in a metal casing worn on a belt resulted in 92.3% (95% confidence interval, CI: 88.9–94.6%). landing protection from female mosquitoes in the afternoon and 68.8% (95% CI: 41.7–83.3%) protection in the evening compared to short untreated clothing. PMD was protective both when combined with short permethrin-treated overalls (afternoon, 68.2%, 95% CI: 52.6–78.7%; evening, 52.3%, 95% CI: 33.8–65.7%) and when used in combination with short untreated overalls (afternoon, 55.0%, 95% CI: 41.7–65.2%; evening, 25.2%, 95% CI: 9.4–38.2%). Whilst long permethrin-treated overalls were protective (afternoon, 61.1%, 95% CI: 51.4–68.8%; evening, 43.0%, 95% CI: 25.5–56.4%), short permethrin-treated overalls and long untreated overalls were not. Exposure to new permethrin-treated fabric in cone tests resulted in 25.0% (95% CI, 17.8–32.2%) and 26.2% (95% CI 16.7–35.8%) mortality for susceptible *Ae. albopictus* and susceptible *Ae. aegypti*, respectively*.* There was a loss of efficacy of permethrin-treated clothing after use in the field, with 3 min knockdown rates of *Ae. albopictus* and 1 h knockdown of *Ae. aegypti* decreasing over time. Participants considered all treatments acceptable.

**Conclusions:**

The portable mosquito coils were highly protective against outdoor biting mosquitoes, although there are safety concerns related to its use. The combination of permethrin-treated clothing and PMD repellent represent an alternative treatment for protection against outdoor-biting mosquitoes.

**Electronic supplementary material:**

The online version of this article (10.1186/s13071-018-3239-0) contains supplementary material, which is available to authorized users.

## Background

Dengue and malaria are two major mosquito-borne diseases which cause considerable morbidity in Southeast Asia (SEA) [1, 2]. At the same time, diseases such as Zika and chikungunya are becoming more common in the region [3–7]. Substantial transmission of these diseases occurs outdoors [8], where long-lasting insecticide-treated nets (LLINs) and indoor residual spraying (IRS) have limited impact. This is particularly true for rubber plantation workers and for those that enter the forests [8–10].

Personal protection methods may be an effective way of preventing mosquito bites, and subsequently preventing the probability of exposure to mosquito-borne diseases when outdoors. They work by repelling or killing the vector, or by providing a physical barrier between the vector and host [11, 12]. In SEA there are generally three ways people protect themselves from outdoor-biting mosquitoes. First, long-sleeved shirts and long trousers are worn to limit the area of exposed skin. Secondly, topical repellents, such as DEET (*N,N*-diethyl-*m*-toluamide), are used to repel mosquitoes. A recent Cochrane review on malaria prevention by topical repellents found that current data is insufficient to identify if topical repellents prevent clinical malaria or malaria infection [13–15]. The lack of a marked protective effect of topical repellents results from the variable duration of protection of 1–12 hours afforded by repellents, sub-optimal levels of compliance and a limited number of properly designed studies [13–16]. In this study, we used the topical repellent para-menthane-3,8-diol (PMD), an isolate from lemon eucalyptus leaves *Corymbia citriodora* [17]. The PMD repellent is effective at repelling mosquitoes, although it is less persistent than DEET [17]. The additional benefit of PMD is its perceived safety and the possibility of sourcing it locally. Although many studies have shown the effectiveness of PMD to decrease mosquito exposure [17–19] and prevent clinical malaria [20], this is the first study to test the repellent in SEA. Thirdly, mosquito coils in a metal casing are recommended for indoor use by the World Health Organization (WHO) [21]. Although coils represent a one billion dollar industry with evidence that it decreases mosquito bites both indoors and outdoors [18, 22–26], there is insufficient evidence to confidently endorse whether they are protective against mosquito-borne diseases such as malaria [13, 27]. In Lao PDR, rubber workers use portable mosquito coils in the field, where they are inserted in a metal case hung from the waist [28]. However, this portable method has not been evaluated for bite protection in a controlled study before.

Permethrin-treated clothing is not widely available in SEA, limiting its use in the region. We have included permethrin-treated clothing in our study, as it is protective against a wide range of mosquito species [29–32], it is highly accepted by the population [33] and has been used by commercial company workers and militaries for decades [34]. Only a few studies have been conducted to evaluate the protection against clinical malaria and dengue, with some evidence that permethrin-treated clothing is protective [34, 35]. The persistence of permethrin is low when the clothing is washed frequently or when the clothing is exposed to ultraviolet light. A recent review of publications has shown that insecticide-treated clothing may reduce malaria incidence by about 50% in the absence of insecticide-treated nets [13]. The limited number of properly designed studies makes it difficult to generate higher certainty of evidence for insecticide-treated clothing.

Since studies on personal protection methods outdoors are limited, we assessed the protective efficacy and the social acceptability of the PMD topical repellent, mosquito coils in a metal casing worn on a belt, permethrin-treated clothing and a combination of permethrin-treated clothing plus PMD outdoors in Lao PDR.

## Methods

### Study area

This study was conducted in two study sites in Xieng-Ngeun district, Luang Prabang Province, northern Lao PDR, from June to August 2016. Dengue is endemic in the study area, while malaria is not. The first site was located in the secondary forest next to Silalek village (19°37'04.57"N, 102°03'27.67"E) where high densities of *Aedes albopictus*, the local dengue vector, occur in the afternoon from 12:00 to 18:00 h [36]. The second site was adjacent to the primary school of Thinkeo village (19°41'08.27"N, 102°07'12.99"E), where putative malaria vectors were collected in the evening from 17:00 to 23:00 h [37]. The *Ae. albopictus* population in our study area is resistant to DDT and malathion, and susceptible to deltamethrin and permethrin [38]. The insecticide resistance status of malaria vectors in the area is not known, although in 2015 in the provincial capital Luang Prabang, located 50 km from the study area, suspected resistance of *Anopheles maculatus* (*s.l.*) to permethrin was identified [39].

### Entomological comparison study

#### Personal protection methods

Participants in the study wore long-sleeved beige cotton overalls. They were provided with clean trousers and tee shirts to wear underneath the overalls. The overall pant legs were either left long or cut just above the knee. The following commercially available treatments were compared using a replicated Latin square design: (i) permethrin-treated overalls (existing marketed treated fabric, 0.052 mg/m^2^, Insectshield®, Insect Shield Llc, Greensboro, USA) with short pant legs; (ii) permethrin-treated overalls with long pant legs; (iii) untreated overalls (Insectshield®) with short pant legs and 19.2% PMD topical repellent (dose of active ingredient (a.i.) in existing marketed PMD product, Citriodiol®, Care Plus, Almere, Netherlands) applied on the lower legs; (iv) permethrin-treated overalls with short pant legs and PMD topical repellent applied on the lower legs; (v) untreated overalls with short pant legs and metofluthrin coils (dose of a.i. in marketed coil products 0.015% a.i., Fumakilla Ltd, Bangkok, Thailand) in a portable metal casing worn on a belt (Additional file 1: Figure S1 shows this in more detail); and (vi) untreated overalls with long pant legs. Typically, adult men wear short-sleeved shirts and shorts in the study area. The use of personal protection methods, such as wearing long trousers, are not common. Untreated long-sleeved overalls with short pant legs therefore served as the control. The permethrin-treated and untreated clothing was not washed throughout the study period. The Insectshield® label claims the protection of the permethrin-treated overalls remains effective for 70 washings. The topical repellent PMD was sprayed 15 cm away from the lower legs by the participants according to manufacturer’s specifications at the start of each day’s collection period (12:00 h in Silalek and 17:00 h in Thinkeo). The spraying was done at a rate of 0.5 ml (3 sprays) per lower leg (lower legs surface area 1.000 cm^2^) in accordance with local customs. The product was applied as recommended by the label sparingly, carefully and evenly to the uncovered skin. More frequent application was not recommended. The administration of repellent was three times lower than WHO guidelines for efficacy testing of mosquito repellents for human skin. This guideline was not used as it did not reflect the local customs nor the label recommendations [40]. The label claims that the product decreases mosquito bites for up to 6 h. Untreated overalls went through the same treatment procedure (i.e. a United States Environmental Protection Agency approved factory dipping process using specific binders) as the treated overalls, without the active ingredient, to provide a direct comparison. The different treatments of the overalls were blinded for the participants. Overalls were stored in a cool, dark place when not in use.

#### Field and laboratory work

To compare the protective efficacy of six treatments against outdoor-biting mosquitoes, human landing catches were carried out from June to August 2016 [41]. Catches were made from 12:00 to 18:00 h in Silalek forest and from 17:00 to 23:00 h in Thinkeo village, periods when exposure to mosquitoes was high and villagers were active outdoors [36].

From both study sites 14 local men 18–39 years-old participated in the study. They only collected mosquitoes from the study area in which they resided, either Thinkeo or Silalek. Each participant undertook a health check. Those with an active infection or a history of malaria and/or dengue were excluded. Before commencing the study, participants wore a piece of the permethrin-treated fabric on their wrist for a week and were sprayed with the repellent on their arms. None showed sensitivity to the insecticides used.

Each study used a 14 × 14 Latin square design to provide a balanced design. During each collection period of 6 h, 14 participants tested six treatments and one control (i.e. two participants simultaneously tested one method). The 14 participants were seated 10 m apart in two straight lines, each of seven people. The two straight lines were located 50 m apart. Each of the 14 participants in one study area was randomly allocated to one position for the duration of the study [42], so that the variation in attractiveness between individuals was associated with the relative attractiveness of the geographical position. Every collection day, the 14 participants were assigned one of the seven different treatments, which was randomized before the start of the study. This resulted in each treatment used twice by every participant throughout one Latin square design (14 collection days). Additional file 2: Table S1 shows the treatment allocations in more detail.

Collectors captured all mosquitoes landing on the exposed or covered lower legs using an aspirator, with those working in darkness wearing a head torch. Mosquitoes were collected in a cup covered with netting, labelled with participant number and collection hour. Collectors sat on a small chair underneath a small plastic cover, to protect them from precipitation and the sun. Every hour, mosquito collections were undertaken for 45 min with a 15 min break. Two supervisors assisted the collectors and checked the protocol was followed. Temperature and relative humidity (RH) were measured in the field using data-loggers (HOBO Pro Onset Computer Corporation, model H08-031-08, Onset Computer Corporation, Cape Cod, USA) placed in the shade 1.80 m above the ground. After collection, the cups with mosquitoes were transported back to the field station, where they were frozen at -20 °C. Mosquitoes were identified to species using a stereomicroscope and identification keys from Thailand [43–46].

#### Sample size consideration

The study was designed to detect a protective efficacy of at least 50% during the afternoon and at least 80% during the evening. The sample size was based on data collected in 2014, where the mean number of mosquitoes collected was 16.41 (± standard deviation, SD 8.49) for the afternoon catches, and a mean of 1.97 ± 2.69 for the evening catches (assuming 80% power and 5% significance level). For afternoon catches, 28 replicates of each treatment, equivalent to a 14 × 14 Latin square, were required. For evening catches 56 replicates, equivalent to two 14 × 14 Latin square designs, were required.

### Cone tests

World Health Organization cone tests were conducted in the laboratory to determine the knockdown (KD) and mortality rate of pyrethroid susceptible *Ae. albopictus* and *Ae. aegypti* strains exposed to permethrin-treated and untreated overalls [47]. Permethrin-treated and untreated overalls, either new or used in the field for two and four weeks, were tested. As controls, cotton shirts, similar to the ones used as undergarments in the study, were used. These controls were run against the fabric of the overalls during every cone test. This experiment was done to determine the potential loss of efficacy of permethrin-treated clothing after use in the field.

Four overalls were randomly selected from each type of overall i.e. permethrin-treated and untreated clothing directly from the factory, used in the field for two weeks and used in the field for four weeks. From each of the 24 overalls a 25 cm^2^ piece of fabric was cut for the cone tests. From the cotton shirts 25 cm^2^ pieces of fabric were also cut for control exposures. The pieces of fabric were secured with several binder clips on a thick plastic sheet covered with aluminium foil. Four cones were placed on top of the fabrics and secured by a second thick sheet of plastic covered with aluminium foil, which had four holes to secure the cones. This was again secured with binder clips and placed on a 45° slanted metal table. Batches of 5 unfed 2–5 days-old female mosquitoes were exposed in the four cones [47]. One of the four cones always served as a control using fabric from the cotton shirts. Female mosquitoes were exposed for 3 min, transferred to a holding cup covered by netting and left for 24 h with access to 10% sugar-solution. Knockdown was measured after exposure and after 1 h. Mortality was recorded after 24 h [47]. If controls showed > 10% mortality per exposure period, the experiment was repeated. On each piece of fabric a total of 60 mosquitoes were exposed (i.e. 5 mosquitoes × 3 cones × 4 replicates). This totalled 240 mosquito exposures for each fabric type (60 mosquitoes × 4 overalls). Bioassays were carried out at 26 ± 2.5 °C and 65 ± 15% RH.

The pyrethroid-susceptible *Ae. albopictus* and *Ae. aegypti* used for the cone tests were reared in our laboratory at 25 ± 2 °C and 80% RH with a 12:12 h photoperiod. The *Ae. albopictus* IPL strain was established in July 2015 from Phomphao village, Luang Prabang Province and Souanmone village, Vientiane Province in Lao PDR. They have been maintained in the laboratory since then. The *Ae. aegypti* mosquitoes were the USDA reference strain from Kasetsart University Bangkok, Thailand [48]. The USDA strain has been reared in the laboratory since its arrival in Lao PDR in July 2014. Susceptibility status of the different strains were confirmed using WHO tube tests with 0.05% deltamethrin and 0.25% permethrin. The *Ae. albopictus* IPL strain was tested before the study in November 2015, when 100% knockdown after 1 h and 100% mortality was noted for both insecticides (*n* = 148 for permethrin, *n* = 178 for deltamethrin). The USDA strain was tested before the study in March 2016 and after the study in October 2016. In March 2016, 100% knockdown after 1 hour and 100% mortality was noted for both insecticides (*n* = 200 for permethrin, *n* = 200 for deltamethrin). Similarly, in October 2016, 100% knockdown after 1 h and 100% mortality was noted for both insecticides (*n* = 175 for permethrin, *N* = 175 for deltamethrin).

### Questionnaire for study participants

We explored the perceptions, attitudes and practices of the mosquito collectors to the different treatments tested using a questionnaire survey one week after the end of collections. Questionnaires were anonymised with no sensitive information collected. Participants were asked to rate the different methods using a score from one to seven to assess their ease of use, protection against mosquitoes and overall preference. Additionally, we asked them questions regarding their willingness to purchase the personal protection methods.

### Data analysis

Mosquito count data were analysed using generalized estimating equations (GEE) with a negative binomial model with log-link function to estimate the difference in landing rates between the treatments and control (IBM SPSS statistics, version 20). The variable mosquito catch was the dependent variable and day number, location of collection and treatment type were covariates included in the model. Protective efficacy was calculated from the odds ratio (OR) using the following formula:

$$ Protective\ efficacy\ \left(\%\right)=\left(1-\left( Odds\ ratio\right)\right)\times 100 $$

Sub-analyses were done for the dominant vector species and putative malaria vectors. For the cone tests, univariate analysis of variance (ANOVA) was used to identify differences in knockdown and mortality between clothing. The dependent variables were 3 min KD, 1 h KD and 24 h mortality, with fabric treatment and exposure day the fixed factors. Comparisons between predicted means for each treatment type were performed using a least significant difference (LSD) *post-hoc* test with the significance level set at 0.001% (5 divided by the 48 tests performed). Data from mosquito collector questionnaires were managed in Excel® 2013 to identify trends and themes.

## Results

### Comparison study

A total of 12,933 female mosquitoes belonging to nine genera and 79 species were collected during the study. The most abundant species were *Culex vishnui* (*s.l.*) (*n* = 5755) and *Ae. albopictus* (*n* = 3025). The putative malaria vectors *An. barbumbrosus* (*s.l.*) (*n* = 233), *An. minimus* (*s.l.*) (*n* = 18), *An. barbirostris* (*s.l.*) (*n* = 14), *An. dirus* (*s.l.*) (*n* = 4), *An. maculatus* (*s.l.*) (*n* = 4), *An. epiroticus* (*n* = 3) and *An. umbrosus* (*n* = 1) made up 2.1% of mosquitoes collected (277/12,933). During the afternoon the average temperature was 27.0 °C (95% CI: 26.8–27.1 °C) and RH 88.4% (95% CI: 87.7–89.0%), whilst in the evening the average temperature was 25.5 °C (95% CI: 25.4–25.6 °C) and RH 96.4% (95% CI: 96.2–96.6%).

### Protective efficacy

**Fig. 1:**
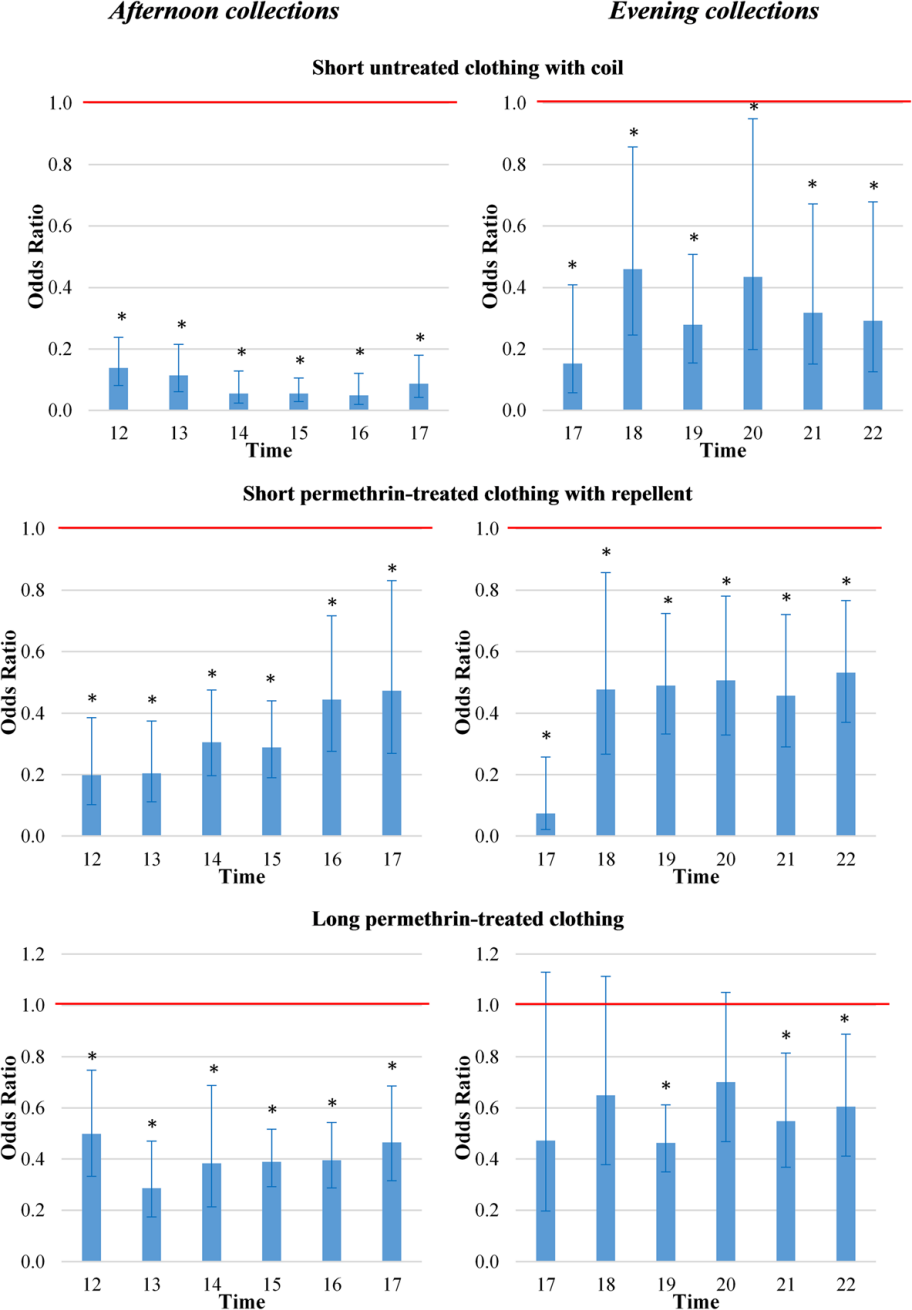
Odds ratios of short untreated clothing with coil, short permethrin-treated clothing with repellent and long permethrin-treated clothing against female mosquitoes landing on exposed legs. Error bars are 95% confidence intervals. The red line highlights odds ratio (OR) = 1. Afternoon collections were undertaken from 12:00 to 18:00 h in the secondary forest of Silalek village. The evening collections were undertaken from 17:00 to 23:00 h at the primary school of Thinkeo village. *Significantly different from short untreated clothing, *P* < 0.05

**Fig. 2:**
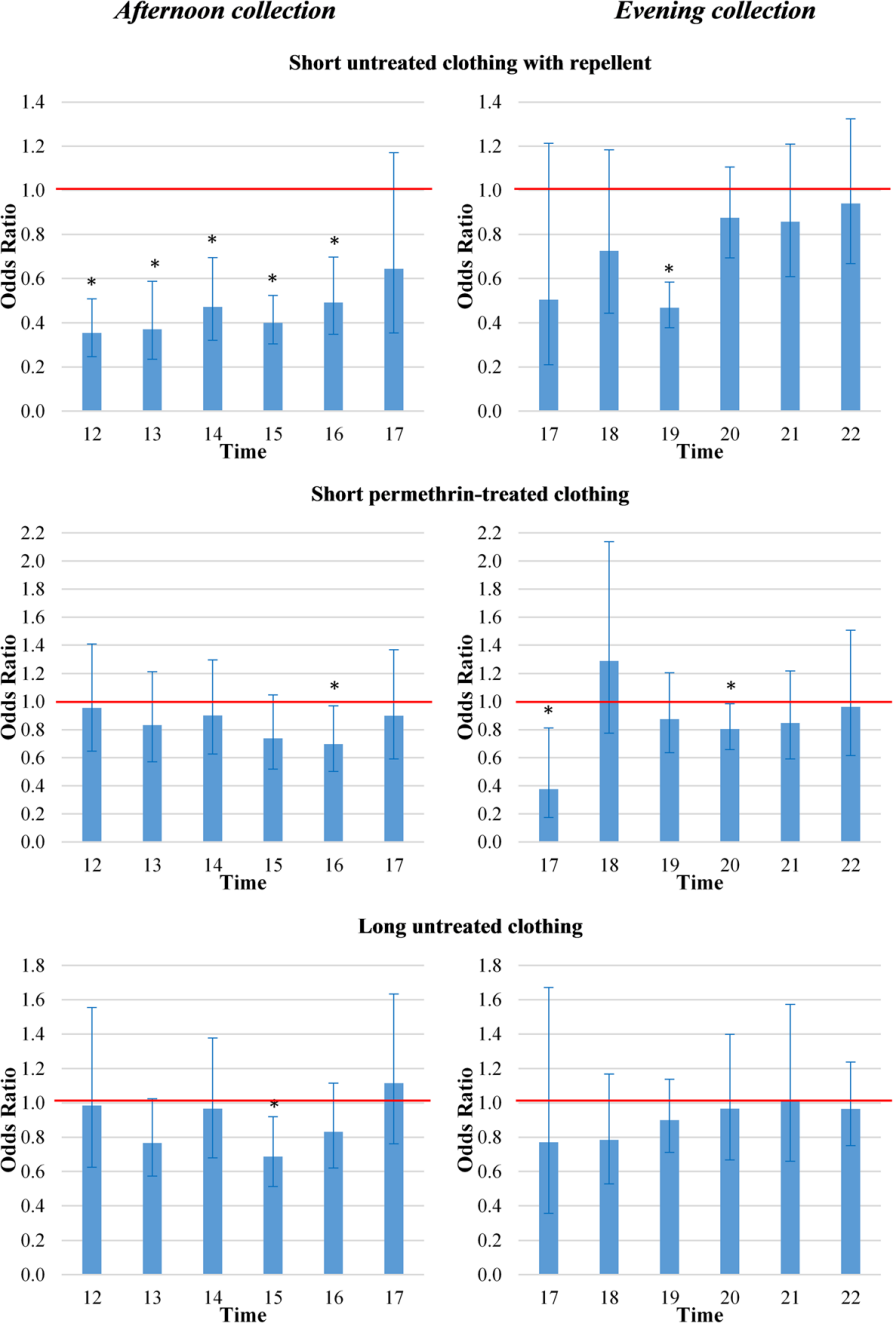
Odds ratios of short untreated clothing with repellent, short permethrin-treated clothing and long untreated clothing against female mosquitoes landing on exposed legs. Error bars are 95% confidence intervals. The red line highlights odds ratio (OR) = 1. Afternoon collections were undertaken from 12:00 to 18:00 h in the secondary forest of Silalek village. The evening collections were undertaken from 17:00 to 23:00 h at the primary school of Thinkeo village. *Significantly different from short untreated clothing, *P* < 0.05

Wearing short untreated clothing with a mosquito coil in a metal casing worn on a belt, using PMD repellent, wearing long permethrin-treated overalls, or a combination of permethrin-treated overalls plus PMD were all protective against mosquitoes in the afternoon and evening compared to wearing untreated clothing (Figs. 1, 2, Table 1). Overall, mosquito coils in a metal casing worn on a belt provided 92.3% protection against all mosquito species during the afternoon (GEE, *df* = 1, *χ*^2^ = 281, *P* ≤ 0.001) and 68.8% during the evening (GEE, *df* = 1, *χ*^2^ = 142, *P* ≤ 0.001) with hourly OR ranging between 0.05 and 0.14 in the afternoon (all *P* ≤ 0.001) and between 0.15 and 0.46 in the evening (all *P* ≤ 0.015; Table 1). Protection was provided throughout the 6 h study period in both the afternoon and evening (Fig. 1). The combination of permethrin-treated clothing plus PMD resulted in 68.2% protection in the afternoon (GEE, *df* = 1, *χ*^2^ = 92, *P* ≤ 0.001) with hourly OR ranging between 0.20 and 0.47 (all *P* ≤ 0.009) and 52.3% in the evening (GEE, *df* = 1, *χ*^2^ = 67, *P* ≤ 0.001) with hourly OR ranging between 0.07 and 0.53 (*P* ≤ 0.013; Table 1). Protection was provided throughout the 6 h study period (Fig. 1). When untreated overalls were used in combination with PMD, this resulted in 55% protective efficacy, with protection for the initial 5 h. During the evening, protective efficacy for the same treatment was only 25.2%, with high variability in protection throughout the collection period (Fig. 2). Long permethrin-treated clothing resulted in 61.1% protection during the afternoon (GEE, *df* = 1, *χ*^2^ = 53, *P* ≤ 0.001), providing protection throughout the 6 h study period. Hourly OR ranged between 0.29 and 0.50, all *P* ≤ 0.001; Fig. 1). Although on average 43.0% protection was measured in the evening (GEE, *df* = 1, *χ*^2^ = 47, *P* ≤ 0.001), protective efficacy was not consistent through time (Fig. 1). Short permethrin-treated clothing and long untreated clothing did not provide any protection from mosquitoes landing on the lower legs (Fig. 2, Table 1).

**Table 1:** Protective efficacy of personal protection methods compared to short untreated clothing for all female mosquito species

Time	Clothing and treatment	Total no. collected	Average collections per collection day (95% CI)	Protective efficacy (95% CI)	*P*
Afternoon	Short permethrin-treated	1331	47.5 (33.3–61.8)	19.3 (3.0–36.8)	0.085
	Long permethrin-treated	593	21.8 (14.5–27.9)	61.1 (51.4–68.8)	<0.001*
	Short untreated + repellent	664	23.7 (16.1–31.3)	55.0 (41.7–65.2)	<0.001*
	Short permethrin-treated + repellent	421	15.0 (10.3–19.8)	68.2 (52.6–78.7)	<0.001*
	Short untreated + coil	138	4.9 (2.5–7.4)	92.3 (88.9–94.6)	<0.001*
	Long untreated	1396	49.9 (36.3–63.4)	14.2 (9.0–32.5)	0.209
	Short untreated	1557	55.6 (39.8–71.4)	1	
Evening	Short permethrin-treated	1187	21.2 (16.4–26.0)	12.6 (-14.6–33.4)	0.329
	Long permethrin-treated	809	14.4 (10.0–18.9)	43.0 (25.5–56.4)	<0.001*
	Short untreated + repellent	1039	18.6 (12.8–24.3)	25.2 (9.4–38.2)	0.003*
	Short permethrin-treated + repellent	707	12.6 (8.4–16.9)	52.3 (33.8–65.7)	<0.001*
	Short untreated + coil	499	8.9 (4.5–13.4)	68.8 (41.7–83.3)	<0.001*
	Long untreated	1233	22.0 (16.2–27.9)	7.3 (-14.7–25.1)	0.484
	Short untreated	1359	24.3 (18.7–29.8)	1	

*Notes*: Results are shown using generalized estimating equations and 95% confidence interval (CI). Afternoon collections were undertaken from 12:00 to 18:00 h in the secondary forest of Silalek village. The evening collections were undertaken from 17:00 to 23:00 h at the primary school of Thinkeo village

*P < 0.05

Similar results were obtained when only *Ae. albopictus* was considered during the afternoon, since it represented 94% of mosquitoes collected (2846/3025). Compared to short untreated clothing the protective efficacy of short untreated clothing with mosquito coil was 91.7%, the combination of permethrin-treated clothing plus PMD was 61.6%, long permethrin-treated clothing was 54.0% and PMD use was 46.1% (Table 2). Short permethrin-treated clothing and long untreated clothing did not provide any protection from *Ae. albopictus* mosquitoes landing on the lower legs. During the evening collections *Cx. vishnui* (*s.l.*) was the most common mosquito species, representing 83.9% of all captured mosquitoes (5737/6833). Against *Cx. vishnui*, the protective efficacy of short untreated clothing with mosquito coil was 70.9%, the combination of permethrin-treated clothing plus PMD was 54.3%, long permethrin-treated clothing was 42.8% and PMD use was 22.5% (Table 2). A total of 273 putative *Anopheles* malaria vectors were collected during the evening and four during the afternoon. Compared to the short untreated clothing, none of the protection methods were protective against putative *Anopheles* malaria vectors during the evening (Table 2).

**Table 2:** Protective efficacy of personal protection methods compared to short untreated clothing for the dominant mosquito species *Ae. albopictus* and *Ae. aegypti*, and putative malaria vectors

Dominant species	Clothing and treatment	Total no. collected	Average collections per collection day (95% CI)	Protective efficacy (95% CI)	*P*
*Ae. albopictus* (afternoon)	Short permethrin-treated	627	22.4 (14.0–30.8)	14.6 (-8.1–32.6)	0.190
	Long permethrin-treated	275	9.8 (6.2–13.5)	54.0 (39.7–64.9)	<0.001*
	Short untreated + repellent	329	11.8 (7.6–15.9)	46.1 (27.9–59.7)	<0.001*
	Short permethrin-treated + repellent	226	8.1 (5.0–11.1)	61.6 (45.5–73.0)	<0.001*
	Short untreated + coil	60	2.1 (0.9–3.4)	91.7 (87.2–94.6)	<0.001*
	Long untreated	657	23.5 (15.0–31.9)	4.4 (-23.3–25.9)	0.728
	Short untreated	672	24.0 (16.4–31.6)		
*Cx. vishnui* (*s.l.*) (evening)	Short permethrin-treated	1004	17.9 (13.3–22.5)	12.0 (-19.8–35.3)	0.417
	Long permethrin-treated	681	12.1 (7.9–16.4)	42.8 (21.6–58.3)	0.001*
	Short untreated + repellent	876	15.6 (10.6–20.7)	22.5 (4.6–37.1)	0.016*
	Short permethrin-treated + repellent	586	10.5 (6.5–14.5)	54.3 (34.6–68.1)	<0.001*
	Short untreated + coil	410	7.3 (3.1–11.5)	70.9 (39.0–86.1)	0.001*
	Long untreated	1045	18.7 (13.2–24.1)	6.5 (-21.4–27.9)	0.615
	Short untreated	1135	20.3 (15.2–25.4)	1	
Putative malaria vectors^a^ (evening)	Short permethrin-treated	44	0.8 (0.4–1.1)	-12.5 (-89.6–33.2)	0.658
	Long permethrin-treated	33	0.6 (0.3–0.9)	23.2 (-13.9–48.3)	0.189
	Short untreated + repellent	55	1.0 (0.4–1.6)	-27.3 (-116.8–25.2)	0.374
	Short permethrin-treated + repellent	28	0.5 (0.3–0.7)	29.3 (-11.6–55.1)	0.137
	Short untreated + coil	32	0.6 (0.3–0.9)	20.2 (-38.8–54.1)	0.425
	Long untreated	42	0.8 (0.4–1.1)	-6.0 (-57.3–28.6)	0.773
	Short untreated	39	0.7 (0.4–1.0)	1	

*Notes*: Results are shown using generalized estimating equations and 95% confidence interval (CI). Afternoon collections were undertaken from 12:00 to 18:00 h in the secondary forest of Silalek village. The evening collections were undertaken from 17:00 to 23:00 h at the primary school of Thinkeo village

^a^Putative malaria mosquitoes *An. barbumbrosus *(*s.l*.), *An. barbirostris *(*s.l*.), *An. dirus *(*s.l.*), *An. maculatus *(*s.l.*) and *An. minimus* (*s.l.*)

*P < 0.05

### Cone tests

There was a decrease in the KD rate of susceptible *Ae. albopictus* exposed to permethrin-treated clothing after use in the field (Table 3). Compared to new permethrin-treated clothing, the KD rate of susceptible *Ae. albopictus* was 79.0% lower when used for two weeks in the field (KD_new_ 13.8% *vs* KD_2 weeks_ 2.9%, LSD *post-hoc* test, *P* < 0.001) and 75.4% lower when used for four weeks in the field (KD_new_ 13.8% *vs* KD_4 weeks_ 3.4%, LSD *post-hoc* test, *P* < 0.001). The 1 h KD of *Ae. albopictus* exposed to new permethrin-treated clothing was 40.4% and mortality was 25.0%. The 1 h KD and mortality of the new clothing did not differ from permethrin-treated clothing used for two weeks nor when used for four weeks in the field (*P* = ns).

**Table 3:** Standardised WHOPES cone tests for permethrin-treated (0.52%) and untreated fabric, both before and after use in the field

Mosquito species	Fabric treatment	Before/after fieldwork^a^	Total no. exposed	KD after exposure (95% CI)	KD 1 h after exposure (95% CI)	Mortality after 24 h (95% CI)
*Aedes albopictus* IPL strain	Untreated	New	239	0.8 (-0.3–2.0)	0	2.5 (-0.1–5.1)
		After (2 weeks)	242	0	0	1.6 (0–3.3)
		After (4 weeks)	239	2.9 (1.4–4.4)	8.1 (5.7–10.6)	2.5 (1.1–3.9)
	Permethrin-treated	New	240	13.8 (7.9–19.6)	40.4 (32.1–48.8)	25.0 (17.8–32.2)
		After (2 weeks)	238	2.9 (0.8–5.1)	43.3 (36.9–49.6)	26.5 (20.8–32.1)
		After (4 weeks)	239	3.4 (1.1–5.6)	44.8 (38.4–51.1)	30.1 (24.3–36.0)
*Aedes aegypti* USDA strain	Untreated	New	240	0	0	0.4 (-0.4–1.3)
		After (2 weeks)	240	0	0	0.4 (-0.4–1.2)
		After (4 weeks)	241	0	0	0.8 (0.0–2.0)
	Permethrin-treated	New	238	4.5 (0.4–8.5)	71.3 (63.6–78.9)	26.2 (16.7–35.8)
		After (2 weeks)	240	5.8 (2.8–8.8)	46.3 (39.9–52.6)	20.8 (15.7–26.0)
		After (4 weeks)	239	7.5 (4.2–10.9)	31.4 (25.5–37.3)	25.1 (19.6–30.6)

*Note*: Results are shown with 95% confidence interval (CI). KD is the knockdown three minutes after exposure, KD 1 h is the knockdown 1 h after exposure

^a^Two weeks of fieldwork were undertaken in Silalek village for afternoon comparisons and four weeks of fieldwork were undertaken in Thinkeo village for evening comparisons

There was a decrease in the 1 h KD rate of susceptible *Ae. aegypti* exposed to permethrin-treated clothing after use in the field compared to new permethrin-treated clothing, the 1 h KD rate of *Ae. aegypti* was 35.1% lower when used for two weeks in the field (1hKD_new_ 71.3% *vs* 1hKD_2 weeks_ 46.3%, LSD *post-hoc* test, *P* < 0.001) and 56.0% lower when used for four weeks in the field (1hKD_new_ 71.3% *vs* 1hKD_4 weeks_ 31.4%, LSD *post-hoc* test, *P* < 0.001). The KD of new permethrin-treated clothing was 4.5% and mortality 26.2%. The KD and mortality rate did not differ between new and used clothing (*P* > 0.05; Table 3).

### Questionnaire for study participants

All 28 mosquito collectors were aware of the risk of mosquito-borne diseases. They confirmed the necessity to protect themselves from mosquitoes, particularly in the forests (26 mentions), rubber plantations (21 mentions) and farms (18 mentions). Less than half of respondents, however, mentioned the use of personal protection methods (11/28), with non-users explaining why they did not use these methods, citing “no money” (*n* = 8), “don’t know how to use” (*n* = 4), “don’t know where to buy” (*n* = 3) and “shop is too far” (*n* = 2). In general, the permethrin-treated clothing was positively reviewed. Although the permethrin-treated clothing does not have any detectable odour, one participant mentioned that the bad smell of the permethrin-treated clothing gave him a headache and made him nauseous. The smoke of the mosquito coils bothered six collectors. Even with the smoke nuisance, the short untreated clothing with coil in a metal casing worn on a belt was popular amongst participants. The mosquito coils were popular due to the clear decrease in mosquito nuisance experienced by participants, the low costs and their familiarity to the product. Other methods popular amongst participants were the long permethrin-treated clothing and the combination of permethrin-treated clothing plus PMD (Additional file 1: Figure S1). Participants were willing to spend an average of 32.000 Lao kip (3.90 USD) per month on personal protection methods with a minimum of 10.000 kip (1.20 USD) and maximum of 150.000 Lao kip (18.10 USD) mentioned. When the costs of the different protection methods were revealed (permethrin-treated clothing 74 USD per overall, bottle of repellent 5 USD and mosquito coils 0.35 USD per coil), this changed the preference of products for most participants (22/28). Nineteen of the participants mentioned only wanting to use repellents and portable mosquito coils for protection, with the remaining three participants only wanting to use the portable mosquito coils.

## Discussion

In this study we compared several commercially available personal protection methods outdoors in rural northern Lao PDR. We found that the protective efficacy of coils in a metal casing worn on a belt was 92% for all female mosquitoes collected from the secondary forest during the afternoon, with a similar 92% protection when only the most dominant vector species *Ae. albopictus* was taken into account. In the village in the evening, protection was 69% for all female mosquitoes collected, with the mosquito coils providing 71% protection against the most dominant vector species *Cx. vishnui* (*s.l.*). Studies on their use as a personal protection method are limited [24, 25]. As far as we know, the use of a portable insecticide coil is novel and this is the first time their use has been tested against outdoor-biting mosquitoes in northern Lao PDR. Despite six collectors complaining about the smoke from the coils, they were popular amongst participants. Mosquito coils are recommended for indoor use by the WHO [49]. However, there is concern that regular use of the coils can increase the risk of lung cancer [18, 50, 51]. One study showed that one mosquito coil emits particulate matters similar to burning 75 to 137 cigarettes. The coils contain carcinogens, with indoor use often exceeding health quality standards [51]. The health effects also include the risk of burns, with the smouldering coil placed close to the human body when used as a personal protection method. The health effects of coils hanging from a belt need to be investigated further.

The combination of permethrin-treated clothing plus PMD topical repellent resulted in an average of 68% protection in the afternoon and 52% protection in the evening for the total number of female mosquitoes collected. More specifically, in the afternoon the permethrin-treated clothing with PMD provided 62% protection against *Ae. albopictus* and in the evening 54% protection against *Cx. vishnui* (*s.l.*). The protective efficacy of permethrin-treated clothing and PMD topical repellent separately was lower than when methods were combined, supporting the findings of earlier work [29, 52–54]. If compliance is high, the combination of both methods could be an important personal protection method. This combination of methods is specifically useful for populations working in a commercial environment such as the rubber plantations, in which high compliance can be enforced.

Long permethrin-treated clothing reduced the mosquito landing rate of all species by 61% in the afternoon and 43% in the evening. The overalls reduced landing rates of the vector species *Ae. albopictus* with 54% and *Cx. vishnui* (*s.l.*) with 43%. In neighbouring Thailand, treatment of school uniforms with 0.52% permethrin resulted in a 71.4% decrease in number of *Ae. aegypti* mosquitoes in the classrooms (1.4 *vs* 4.9) during the five month study [35]. The evidence that permethrin-treated clothing is protective against mosquito-borne diseases is weak. In the Thai school study, the ability to measure an impact of pyrethroid-treated uniforms to protect against dengue was constrained [35] due to the poor persistence of the insecticide on the frequently washed clothing and the low number of dengue cases during the study. A similar lack of protection was found against malaria in Thai soldiers provided with permethrin-treated uniforms [55]. In contrast, a study in the Colombian military showed 88.6% protection from clinical episodes of malaria [56]. Additionally, a study among refugees in Afghanistan using permethrin-treated chaddars (a veil) and sheets reduced malaria by 64% in children < 10 years and 38% in refugees < 20 years. [57]. Currently, there is no consensus in the scientific community on the protection of permethrin-treated clothing against malaria [34]. The use of permethrin-treated clothing for public health purposes show promise. However, more extensive clinical-based research is necessary to confirm this.

Permethrin-treated clothing straight from the factory resulted in only 25% mortality after exposure of *Ae. albopictus* in cone tests and in only 26% mortality of *Ae. aegypti*. These are both lower than the 80% mortality that the WHO recommends for insecticidal-treated nets [47] and lower than the 97% recorded in another study where *Ae. aegypti* were exposed to the same clothing [31, 58]. Both strains of mosquitoes in our study were confirmed susceptible to pyrethroids. As a result, the 26% mortality suggests that the fabric treatment might not have been successful. Despite the low mortality shown in the cone tests, the permethrin-treated clothing still provided additional protection compared to the untreated clothing. The lack of agreement between cone assays and field findings could be related to the cone tests measuring the mortality effect after exposure to the permethrin-treated clothing, while the field comparison measures the repellence effect.

As insecticide-treated clothing has not been tested in the field in Lao PDR before, our goal was to compare new permethrin-treated clothing with other commonly used personal protection methods. The permethrin-treated clothing was re-used throughout the study period, as it was assumed that not washing the clothing and minimizing ultraviolet exposure would ensure the clothing remained equally protective for the duration of the study time. The cone tests, however, showed some decease in efficacy, with the KD of susceptible *Ae. albopictus* and 1 h KD of susceptible *Ae. aegypti* decreasing after using the permethrin-treated clothing in the field. The efficacy of the permethrin-treated clothing started to wane after only two weeks in the field. Further improvement of the persistence of permethrin in clothing is essential for its success in disease control.

When the topical repellent PMD was applied to the exposed limbs of human subjects wearing short untreated clothing, the number of *Ae. albopictus* and *Cx. vishnui* (*s.l.*) landing declined. Several studies have shown the effectiveness of PMD against dengue and malaria vectors [17–19, 59]. In Bolivia, PMD in combination with permethrin-treated bed nets decreased malaria incidence [20]. Based on previous studies and the current evaluation, the topical repellent PMD is a good personal protection method for afternoon biting *Ae. albopictus* mosquitoes. This biodegradable product does not dissolve synthetics and can easily be synthesized using locally available products at low cost. However, as it is less persistent than DEET, it needs more regular re-application. Although PMD provides protection against mosquitoes, like all other topical repellents, user-compliance may limit the control of disease transmission [13–15, 60]. Currently, topical repellents may only be important for public health purposes when implemented in a commercial environment, where workers are required to use topical repellent before starting work and there is incentive to re-apply it regularly.

We found that covering the lower legs with permethrin-treated clothing can reduce mosquito-landing rates. Protection is, however, only local, as mosquitoes can still find exposed skin on the hands and head which are not protected by the clothing [29]. It is important to understand if the permethrin-treated clothing also protects the exposed skin next to it, a so-called halo effect. In our study, short permethrin-treated clothing did not result in any additional protection of lower legs. This is contrary to a laboratory study in the US, where exposed skin was also protective against mosquito bites when wearing the permethrin-treated clothing [29]. Correspondingly, in another laboratory study a similar number of *Ae. aegypti* landed on the arm regardless of whether it was fully or partially covered by permethrin-treated clothing [58]. The halo effect of permethrin-treated clothing may not exist outdoors when there is greater air movement than indoors. More studies are necessary to confirm this.

During our study we used mosquito-landing rates as a proxy for mosquito bites, the gold standard for measuring mosquito exposure in the field. The limitation of using landing rates as a proxy is that it does not measure the impact of the physical barrier: thick clothing. Additionally, landing rates do not take into account the possible difference in behaviour between mosquitoes that land on permethrin-treated and untreated fabric. Mosquitoes have been known to persistently try to bite through untreated clothing, while mosquitoes on permethrin-treated clothing may not stay on the material long enough for a successful bite [29]. An experiment in a free-flight room with the same permethrin-treated clothing showed 24% protective efficacy of *Ae. aegypti* [58]. Biting inhibition was much higher at 91%, due to the permethrin affecting the mosquito motor skills once the mosquitoes landed [58]. It is thus important to note that, although there was no reduction in the number of mosquitoes landing on long untreated clothing, the thick long overalls worn by the participants may have resulted in lower biting rates compared to the short untreated trousers.

A limitation of this study is the 10 m distance used between treatments in the field comparisons, which is lower than the ≥ 20 m recommended by the WHO [40]. The 10 m distance may not have been sufficient to avoid diversion of mosquitoes between the different treatments. One treatment may have pushed mosquitoes towards the neighbouring participants, which may have enhanced the mosquito numbers collected in the controls. This could have affected the protective efficacy. Another limitation was that only 277 putative malaria vectors were collected during this study. Better-designed studies are essential to identify personal and community protection of personal protection methods for the local populations in SEA, including studies on the efficacy of these methods against malaria vectors. Other personal protection methods should also be considered, such as metofluthrin emanators and repellent anklets [61, 62]. Further studies are also needed to determine why compliance of personal protection methods is low and how this can be improved.

The questionnaire was not designed to evaluate the safety or social acceptance of treatments used at scale. However, these initial data do show the acceptability of mosquito coils, PMD repellent and permethrin-treated clothing was high. A major barrier for the use of personal protection methods is affordability [63]. Study participants were aware of the necessity to protect themselves from mosquitoes outdoors in the forests, rubber plantations and farms. They did not, however, use personal protection methods often, due to the high costs, lack of knowledge about what treatments could provide protection and accessibility of the products. The PMD repellent and permethrin-treated clothing can be made much more affordable than what was paid for by this study. Unfortunately, high acceptability does not necessarily translate into high compliance, even when the product is offered for free [15]. The use of personal protection methods to provide community protection may only be possible in commercial areas such as tree-plantation estates and the military, where organizations can cover the costs of the interventions and insist on high levels of compliance. The major challenge in the future of personal protection methods will be to conduct well designed studies in a variety of settings.

## Conclusions

Mosquito coils in a metal casing worn on a belt provided greater mosquito landing protection against female *Ae. albopictus* and *Cx. vishnui* (*s.l.*) for six hours, as compared to untreated short overalls. Although the smoke of the mosquito coils was a nuisance for some participants, the protection against mosquitoes outweighed its discomfort in mosquito collectors. These results are encouraging, although more studies need to be undertaken to assess the safety and disease prevention ability of this approach. The combination of permethrin-treated clothing plus PMD could be a good alternative to the mosquito coils. With further improvement of the persistence of permethrin in clothing, both methods can become important tools for public health, especially when products are supplied at affordable costs and there is high user compliance.

## Additional files

Additional file 1: **Figure S1.** Mosquito coil in a portable cage. Example of a mosquito coil in a cage worn on a belt. (TIF 6097 kb)

Additional file 2: **Table S2.** Treatment allocations. The treatments are: permethrin-treated overalls with short pant legs (A), permethrin-treated overalls with long pant legs (B), untreated overalls with short pant legs and PMD topical repellent applied on the lower legs (C), permethrin-treated overalls with short pant legs and PMD topical repellent applied on the lower legs (D), untreated overalls with short pant legs and metofluthrin coils in a portable metal casing worn on a belt (E), untreated overalls with long pant legs (F), and untreated long-sleeved overalls with short pant legs (G). Afternoon collections were undertaken from 12:00 to 18:00 h in the secondary forest of Silalek village. The evening collections were undertaken from 17:00 to 23:00 h at the primary school of Thinkeo village. (DOCX 25 kb)

## Abbreviations

95% CI: 95% Confidence interval
DEET: *N,N*-diethyl-*m*-toluamide
*df*: degrees of freedom
GEE: Generalized estimating equations
IRS: Indoor residual spraying
KD: Knockdown
Lao PDR: Lao People’s Democratic Republic
LLINs: Long-lasting insecticide-treated nets
OR: Odds ratio
PMD: Para-menthane-3,8-diol
RH: Relative humidity
SD: Standard deviation
SEA: Southeast Asia
WHO: World Health Organization
