# Supplementary figures and images for: Field evaluation of personal protection methods against outdoor-biting mosquitoes in Lao PDR
Source: Parasit Vectors. 2018 Dec 17;11:661. doi: 10.1186/s13071-018-3239-0 (PMC6296151; doi:10.1186/s13071-018-3239-0)

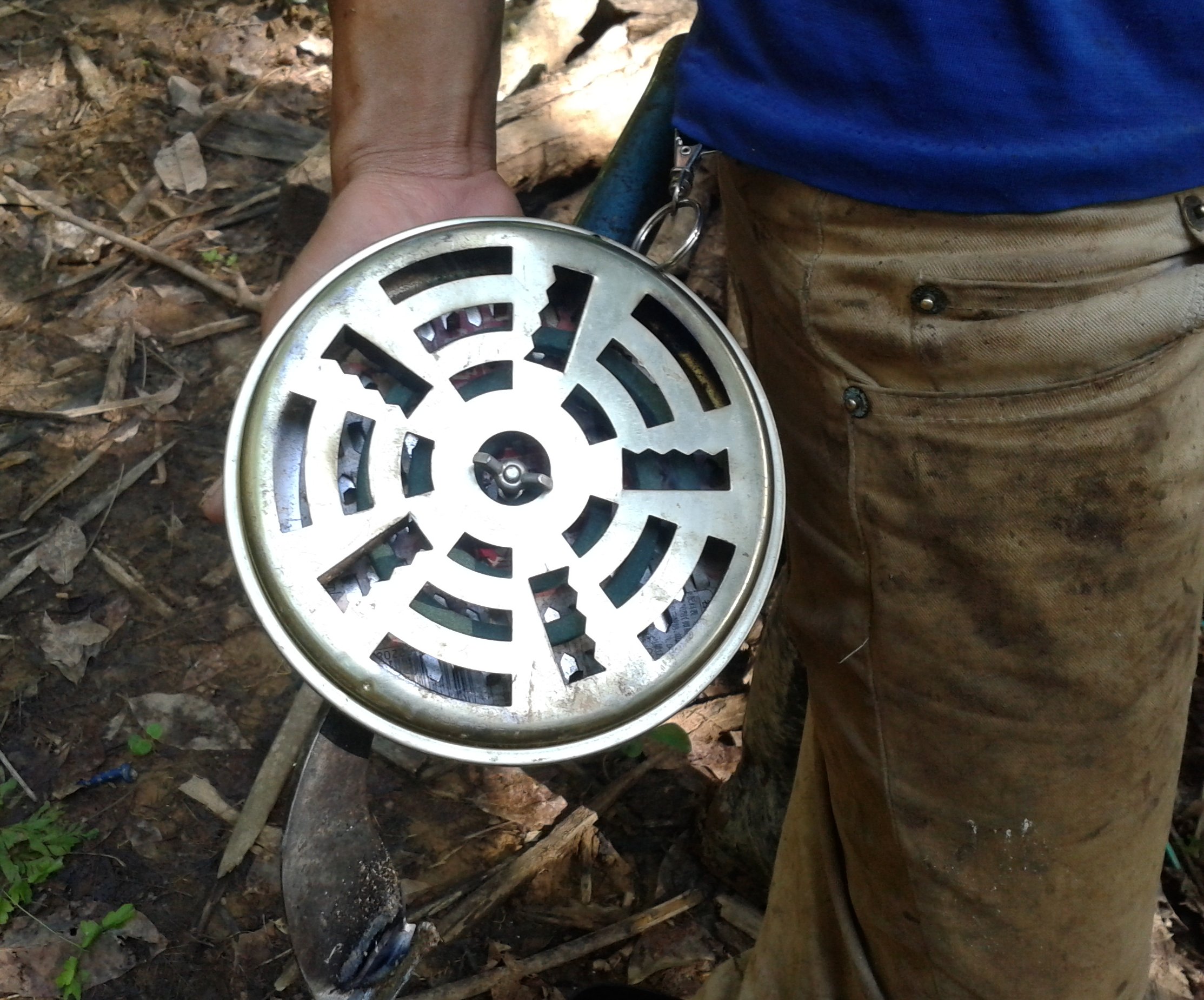

Supplement: Supplementary file 1 — Figure S1. Mosquito coil in a portable cage. Example of a mosquito coil in a cage worn on a belt. (TIF 6097 kb) [file 13071_2018_3239_MOESM1_ESM.tif]
