# Supplementary material for: Field evaluation of personal protection methods against outdoor-biting mosquitoes in Lao PDR
Source: Parasit Vectors. 2018 Dec 17;11:661. doi: 10.1186/s13071-018-3239-0 (PMC6296151; doi:10.1186/s13071-018-3239-0)
Supplement: Supplementary file 2 — Table S2. Treatment allocations. The treatments are: permethrin-treated overalls with short pant legs (A), permethrin-treated overalls with long pant legs (B), untreated overalls with short pant legs and PMD topical repellent applied on the lower legs (C), permethrin-treated overalls with short pant legs and PMD topical repellent applied on the lower legs (D), untreated overalls with short pant legs and metofluthrin coils in a portable metal casing worn on a belt (E), untreated overalls with long pant legs (F), and untreated long-sleeved overalls with short pant legs (G). Afternoon collections were undertaken from 12:00 to 18:00 h in the secondary forest of Silalek village. The evening collections were undertaken from 17:00 to 23:00 h at the primary school of Thinkeo village. (DOCX 25 kb) [file 13071_2018_3239_MOESM2_ESM.docx]

**Additional file 2: Table S1. Treatment allocations**

**Afternoon collections**

| **Location** | **1** | **2** | **3** | **4** | **5** | **6** | **7** | **8** | **9** | **10** | **11** | **12** | **13** | **14** |
| --- | --- | --- | --- | --- | --- | --- | --- | --- | --- | --- | --- | --- | --- | --- |
| **Day 1** | B | D | F | E | G | C | A | E | F | C | D | G | A | B |
| **Day 2** | D | F | E | C | B | A | G | F | A | D | C | E | B | G |
| **Day 3** | F | C | G | A | E | B | D | B | D | E | G | C | F | A |
| **Day 4** | A | G | D | B | F | E | C | G | B | F | A | D | C | E |
| **Day 5** | C | E | A | F | D | G | B | D | G | B | F | A | E | C |
| **Day 6** | G | B | C | D | A | F | E | C | E | A | B | F | G | D |
| **Day 7** | E | A | B | G | C | D | F | A | C | G | E | B | D | F |
| **Day 8** | D | C | B | F | A | E | G | D | A | C | F | B | E | G |
| **Day 9** | G | E | D | C | B | A | F | C | E | D | G | F | B | A |
| **Day 10** | A | D | E | B | G | F | C | F | G | A | B | C | D | E |
| **Day 11** | F | G | C | E | D | B | A | E | B | F | D | A | G | C |
| **Day 12** | B | F | A | G | C | D | E | B | D | G | C | E | A | F |
| **Day 13** | E | B | G | A | F | C | D | G | F | E | A | D | C | B |
| **Day 14** | C | A | F | D | E | G | B | A | C | B | E | G | F | D |

**Evening collections**

| **Location** | **1** | **2** | **3** | **4** | **5** | **6** | **7** | **8** | **9** | **10** | **11** | **12** | **13** | **14** |
| --- | --- | --- | --- | --- | --- | --- | --- | --- | --- | --- | --- | --- | --- | --- |
| **Day 1** | A | E | D | G | C | F | B | A | E | D | G | C | F | B |
| **Day 2** | G | C | F | A | D | B | E | G | C | F | A | D | B | E |
| **Day 3** | E | F | A | B | G | C | D | E | F | A | B | G | C | D |
| **Day 4** | F | G | B | C | E | D | A | F | G | B | C | E | D | A |
| **Day 5** | C | D | G | E | B | A | F | C | D | G | E | B | A | F |
| **Day 6** | D | B | C | F | A | E | G | D | B | C | F | A | E | G |
| **Day 7** | B | A | E | D | F | G | C | B | A | E | D | F | G | C |
| **Day 8** | C | B | A | G | F | D | E | A | C | G | F | E | B | D |
| **Day 9** | B | F | G | A | E | C | D | B | G | A | D | C | E | F |
| **Day 10** | D | E | B | C | A | G | F | C | B | F | A | D | G | E |
| **Day 11** | A | C | E | F | D | B | G | D | E | B | C | A | F | G |
| **Day 12** | F | G | D | E | B | A | C | G | D | E | B | F | A | C |
| **Day 13** | G | D | F | B | C | E | A | E | F | D | G | B | C | A |
| **Day 14** | E | A | C | D | G | F | B | F | A | C | E | G | D | B |
| **Day 15** | F | G | D | E | B | A | C | G | D | E | B | F | A | C |
| **Day 16** | E | B | G | C | F | D | A | D | F | B | A | G | C | E |
| **Day 17** | D | E | F | G | A | C | B | F | G | D | C | B | E | A |
| **Day 18** | G | C | E | A | D | B | F | C | B | A | G | E | F | D |
| **Day 19** | B | F | A | D | C | E | G | B | C | G | E | A | D | F |
| **Day 20** | A | D | C | B | G | F | E | A | E | C | F | D | G | B |
| **Day 21** | C | A | B | F | E | G | D | E | A | F | D | C | B | G |
| **Day 22** | B | E | A | G | D | F | C | B | D | A | F | E | G | C |
| **Day 23** | D | F | C | E | A | G | B | D | A | B | C | F | E | G |
| **Day 24** | C | G | B | D | F | A | E | E | B | G | D | A | C | F |
| **Day 25** | F | B | E | A | C | D | G | C | G | F | A | D | B | E |
| **Day 26** | A | D | G | B | E | C | F | F | C | D | E | G | A | B |
| **Day 27** | E | A | F | C | G | B | D | G | F | E | B | C | D | A |
| **Day 28** | G | C | D | F | B | E | A | A | E | C | G | B | F | D |
